# Creating safer cancer care with ethnic minority patients: A qualitative analysis of the experiences of cancer service staff

**DOI:** 10.1111/hex.13979

**Published:** 2024-01-30

**Authors:** Ashfaq Chauhan, Bronwyn Newman, Elizabeth Manias, Kathryn Joseph, Desiree Leone, Ramesh L. Walpola, Holly Seale, Allan Ben Smith, Reema Harrison

**Affiliations:** ^1^ Centre for Health Systems and Safety Research, Australian Institute of Health Innovation Macquarie University Macquarie Park New South Wales Australia; ^2^ School of Nursing and Midwifery Monash University Clayton Victoria Australia; ^3^ Multicultural Health Services Western Sydney Local Health District North Parramatta New South Wales Australia; ^4^ School of Health Sciences UNSW Sydney Kensington New South Wales Australia; ^5^ School of Population Health UNSW Sydney Kensington New South Wales Australia; ^6^ The Daffodil Centre, The University of Sydney, a joint venture with Cancer Council NSW Camperdown New South Wales Australia; ^7^ Ingham Institute for Applied Medical Research Liverpool UK

**Keywords:** cancer care, consumer engagement, patient safety, SEIPS

## Abstract

**Introduction:**

Effective consumer engagement practices can enhance patient safety. This is important for consumers from ethnic minority backgrounds who are exposed to increased risk of patient safety events. Using the Systems Engineering Initiative for Patient Safety model, this study explored staff experiences of creating opportunities for engagement with consumers from ethnic minority backgrounds to contribute to their cancer care safety.

**Method:**

A qualitative study was conducted using semistructured interviews with cancer service staff from four cancer services across two states in Australia. Purposive sampling was used to recruit healthcare staff from a diverse range of professions. Data were analysed using the Framework Analysis method.

**Results:**

Fifty‐four interviews were conducted with healthcare staff. Analysis of the qualitative interview data identified enablers and associated challenges that contributed to creating a shared understanding between consumers and staff of the information, processes, expectations and problems arising in care. Enablers and challenges are reported in relation to four themes: (1) co‐creating safety through shared understanding of care processes; (2) tools and technologies support planned communication; (3) organisational policy levers exist but lack implementation in direct care and (4) formal tasks incorporate consumer engagement more readily than informal interactions.

**Conclusion:**

The availability of infrastructure and resources to support communication with consumers from ethnic minority backgrounds was limited to specific tasks across the cancer care continuum. Strategies implemented by health services to foster effective communication during formal interactions now require expansion to support and create conditions for effective consumer engagement during informal and everyday care tasks. The use of innovative language support tools and cultural considerations are required at the service and system level to support consumer engagement in all types of care interactions.

**Public and Patient Involvement:**

The study was embedded within a larger project that included a consumer investigator and was guided by a consumer advisory group (CAG). These consumer team members have lived experience of cancer and are from diverse ethnic backgrounds. CAG members provided feedback on the draft interview guide and participant information for this study.

## 1 INTRODUCTION

Traditionally, patient safety has been thought of as an absence of preventable harm during the process of care^1^; but can also be conceptualised as achieving the safest possible care in the available circumstances.^2^ Consumers from ethnic minority backgrounds accessing healthcare are often exposed to a higher risk of patient safety events (events that could have or did result in harm to the patient)^3^ such as medication‐related harm and healthcare‐acquired infections compared to those of nonethnic minority backgrounds.^4^ Many factors contribute to their increased vulnerability to safety events, including a lack of meaningful engagement in their care.^4^ Patient safety for ethnic minority patients is of particular importance in cancer services where the possibility of preventable harm remains high.^5^, ^6^ A retrospective review of 300 medical records of patients from ethnic minority backgrounds accessing two Australian cancer services identified that almost one‐third of the medical records (104/300, 34.6%) had at least one safety event documented.^7^ Medication‐related safety events (medication administration and nonadherence), patient accidents (falls) and patient behaviour (noncompliance) were some of the safety event types identified in the retrospective record review study.^7^, ^8^

Healthcare is a complex sociotechnical system that involves constant interactions between humans, machines, environments, work activities and organisational structures and processes.^9^ Consumers, defined as patients and their family or support person/s,^10^ are an integral part of this sociotechnical system.^11^ The role of family or support person in determining the safety of healthcare for ethnic minority patients has been identified as important^4^, ^12^ and the use of the term ‘consumer’ reflects this. By creating conditions that facilitate consumer engagement in care, consumers can co‐create safer care practices.^13^, ^14^ Consumer engagement, defined as meaningful involvement of consumers in healthcare decision making,^15^ has the potential to reduce errors, increase shared decision‐making and consumer understanding of care, improve health outcomes and lower healthcare costs.^16^ Consumer engagement can act as a feedback loop informing practice and care improvement to enhance patient safety.^11^, ^17^, ^18^ The World Health Organisation Global Patient Safety Action Plan (2021–2030) recognises consumers' engagement in their care as one of the key strategies to reduce harm to the patient and provide safe and respectful care.^19^ Healthcare staff can facilitate consumer engagement by sharing information with consumers about their care and encouraging them to ask questions.^20^, ^21^, ^22^ However, the efficacy of these strategies such as encouraging to ask questions to enhance the safety of consumers from ethnic minority backgrounds, is not known.^23^

In a cancer care system, effective consumer engagement practices can enhance patient safety as consumers can identify and report issues relating to unsafe care.^22^, ^24^, ^25^, ^26^, ^27^ To date, research with consumers from ethnic minority backgrounds has identified various factors influencing consumer engagement across different levels of healthcare decision‐making. At the direct care level, education, experience, language proficiency and diversity of cultural background shape how consumers from ethnic minority backgrounds are engaged in their care.^28^ At the service level, building staff and consumer capacity and providing culturally sensitive care have been highlighted, but there is limited knowledge and understanding of specific mechanisms through which consumer engagement can be realised for consumers from ethnic minority backgrounds.^15^ System‐level policies on the use of professional interpreters may address some of the communication barriers but may not address the nuanced inter‐ and intraethnic variations that exist within ethnic minority groups to facilitate consumer engagement.^28^

Health systems models enable us to consider the factors that impact consumer engagement and safety in cancer services.^10^, ^29^ Delivery of safe care could be enhanced when working with consumers from ethnic minority backgrounds by establishing the various systems and service components that influence engagement. The Systems Engineering Initiative for Patient Safety (SEIPS) model is widely used to explore how health systems and services create safety in care and has been applied in diverse care settings.^29^ Application of the SEIPS model could enhance our understanding of how safety is created and maintained by increasing consumer engagement in care.^11^, ^17^, ^18^ Applying the SEIPS model to understand the experiences of cancer service staff can help identify the conditions that foster consumer engagement for safety. This study aimed to explore staff experiences of creating opportunities for consumer engagement with consumers from ethnic minority backgrounds to contribute to their safety in cancer care. The specific objective was to identify enablers and challenges for co‐creating safety with consumers from ethnic minority backgrounds accessing cancer care in Australia.

## 2 METHODS

This study is reported in accordance with the Consolidated Criteria for Reporting Qualitative Studies guidelines (File A).^30^

### 2.1 Design

A cross‐sectional qualitative descriptive study was conducted using semistructured interviews with cancer service staff.

### 2.2 Setting

Four cancer services, two in the state of New South Wales (NSW) and two in the state of Victoria (VIC) in Australia were selected. VIC and NSW are two of the top three states that have the highest proportion of overseas‐born population.^31^ The four cancer services served a substantial population from diverse ethnic minority backgrounds.^32^ All four cancer services provided chemotherapy, radiotherapy, immunotherapy, haematology and palliative care services across inpatient, day therapy and outpatient cancer care settings in metropolitan areas within large cities.

### 2.3 Recruitment

Purposive sampling was used to recruit a range of eligible cancer service staff (clinicians, managers and administrative staff) across the four services. Eligible participants had worked at the participating cancer service as a permanent employee (full‐time or part‐time) for more than 6 months. Healthcare staff in casual roles were excluded as the nature of casual employment may decrease awareness of service‐specific policies and procedures. Diversity in professional roles was sought to enable the collection of experiences from staff contributing to different aspects of care. The participating site‐specific collaborators distributed study advertisements to staff via their internal networks. Interested staff contacted the research team directly. Eligibility was confirmed by phone or email, and informed consent was obtained before conducting the interviews.

### 2.4 Data collection

Semistructured interviews were conducted by three researchers (A. C.; B. N. and K. J.) either face‐to‐face, online or via telephone. All three researchers (A. C.; B. N. and K. J.) had prior experience of conducting semistructured interviews. Researchers (A. C.; B. N. and K. J.) did not have any professional relationship with the staff interviewed. Interviews took place from March 2021 to September 2022 and lasted between 30 and 50 min. Semistructured interviews were selected as a suitable and flexible approach to data collection to obtain in‐depth data and pursue other interesting lines of enquiry as they arose.^33^ An interview guide was developed and used to conduct the semistructured interviews. The interview guide was developed using the experienced‐based codesign toolkits^34^, ^35^ incorporating the experiences of the research team in conducting qualitative research and feedback from the members of the consumer advisory group associated with the research project that this study was part of. This guide covered three main topics: (i) safety issues that impact consumers from ethnic minorities in cancer care; (ii) staff perceptions and experiences of their ability to effectively engage with consumers from ethnic minorities and (iii) strategies used and support available to staff to enable the engagement of ethnic minority consumers in their care to enhance their safety. After verifying eligibility criteria, a mutually agreeable time was established by the researcher with the potential participant along with confirmation of the mode of the interview (face‐to‐face, via telephone or online). Written consent to participate was obtained before the interview with verbal consent also confirmed at interview commencement. Interviews were recorded using an audio recorder and the recording was stored in a password‐protected secure cloud‐based location accessed only by the research team.

### 2.5 Data analysis

Interview audio recordings were transcribed verbatim by a professional transcriber. Data were both deductively and inductively analysed using a Framework Analysis method grounded in the SEIPS 3.0 Model.^17^, ^36^ The five domains of the SEIPS model; person, task, tools and technology, environment and organisation constituted the elements of the framework for analysis. The data was first deductively analysed and coded under these five categories. Following this, an inductive approach was used and the resulting themes were formed. After transcription, two research fellows with clinical backgrounds—one physiotherapist (A. C.) and one social worker (B. N.), familiarised themselves with the data by rereading the transcripts several times. Following this, 10 transcripts were independently coded by the same two researchers. The two researchers met to compare their findings and develop a working analytical framework. The remaining transcripts were then coded by the two same researchers using the categories and codes in the working analytical framework. This was an iterative process whereby the two researchers met weekly to discuss findings and update the working analytical framework as required. Findings were also discussed with the principal researcher (R. H.) throughout this process in separate weekly meetings and the analytical framework was refined. A Framework Matrix charting the final categories and codes (File B) was developed using Microsoft Excel Spreadsheet. A subset of transcripts was reviewed by other team members (E. M., K. J., R. L. W., H. S., D. L. and A. B. S.) and the findings were discussed as a group. Following this, themes were identified, with preliminary themes defined and refined by the research team. Final themes were checked by all research team members. This process addressed rigour during data analysis. Participant codes were generated with ‘Site#’ representing the respective cancer service and ‘P#’ representing the participant and their unique number followed by the profession category.

## 3 FINDINGS

Fifty‐four interviews were completed with a range of healthcare staff across the four cancer services (15 interviews each at the two cancer services in NSW; 10 and 14 at cancer services in VIC, respectively) reaching the data saturation. Occupations were categorised into administrative, allied health, nursing and medical staff to maintain participants' anonymity. Seventeen nurses (nursing unit managers, care coordinators, nurse leads and clinical nurses), 16 medical staff (oncologists, radiologists and surgeons), 11 administrative staff (reception staff, health managers and operations managers) and 9 allied health staff (social workers, speech pathologists, wellness staff and genetic counsellors) participated.

Analysis of the qualitative interview data revealed that the enablers and challenges to co‐create safety with consumers from ethnic minority backgrounds transcended through various components of the SEIPS model. Enablers and challenges are reported in relation to four themes that were developed with reference to the five components of the SEIPS model: (1) co‐creating safety through consumer‐service provider dyad; (2) tools and technologies support planned communication; (3) organisational policy levers exist but lack implementation in direct care and (4) formal tasks incorporate consumer engagement more readily than informal interactions. Enablers collectively supported the co‐creation of safer care by creating a shared understanding between consumers and staff of the information, processes, expectations and problems arising in care. However, various challenges were also identified whereby these enablers did not fully create the conditions that supported shared understanding. ‘Shared understanding’ was therefore identified as an underpinning theme.

### 3.1 Co‐creating safety through a shared understanding of care processes

Consumer‐service provider dyad as a component of SEIPS model comprised three main categories; (1) person (such as interpreters, bilingual staff and support person(s) that supported this dyad by providing language support, (2) consumers beliefs of cancer and their attitude to engagement and (3) trust and relationship between consumers and respondents. The elements comprising these three categories enabled certain tasks that contributed towards enhancing shared understanding of care processes between consumers from ethnic minority backgrounds and healthcare staff from cancer services.

#### 3.1.1 Person (interpreters, bilingual staff and family/support person)

Interpreters, bilingual staff and family/support persons enabled consumer engagement for co‐creating safety by promoting cross‐cultural communication in various capacities, but challenges were also identified with their use. Interpreters were more readily available and used for tasks that were deemed high risk and planned such as getting informed consent for clinical trials or treatment or procedure related to cancer, first chemotherapy or radiotherapy education session or appointments with oncologists/haematologists. Interpreter use was considered of high importance due to cancer treatment becoming more complicated with new drugs and clinical trials introduced. Interpreter use for these tasks was further enforced through service policies. When compared to these, interpreters were not readily used for unplanned tasks such as everyday care interactions during chemotherapy or radiotherapy treatment sessions, everyday inpatient cancer care interactions, or during ad‐hoc appointments with cancer clinicians. The cumbersome booking system, not being able to source an interpreter in a particular dialect and unplanned and shorter duration of the interactions impacted interpreter booking and usage. Poor cross‐cultural communication during unplanned tasks made it difficult to create shared understanding of care processes potentially leading to safety events. Healthcare staff verbalised that inadequate language support resulted in difficulty in creating common understanding of care processes, such as recognising and responding to side‐effects of cancer‐related medications.

If patients are signing consent forms for a procedure and they're non‐English speaking they have to have an interpreter. (SiteB‐P1_Adminstrative)

So, the actual fine tuning of the medication, for example, may not be effectively done … as you would be aware now there's more complicated treatment schedules and regimens. (SiteB_P3_Nurse)

Just today I've had to … use her daughter and son, because we just couldn't (book the interpreter) ‐ and the phone interpreter wasn't working. (SiteA_P2_Nurse)

There's a lot of different dialects, and sometimes I've had issues where patients have not been of the same dialect in terms of the language. (SiteB_P4_Medical)

When (ethnic minority) patients are on chemotherapy, we want them to ‐ if they have a fever, to come into emergency and we've definitely had patients who haven't done that … and as a result, have had adverse effects overall in the long run. (Site B_P4_Medical)

For some cancer care‐specific interactions where interpreters were used, concerns were raised about interpreters providing incomplete information to consumers and interpreters' comfort with translating some information due to cultural factors (such as when a female interpreter was used to consult with a male patient with prostate cancer).

I had said to her, you really can't afford to lose any more weight. And he interpreted, you must not gain weight. (SiteD_P5_AlliedHealth)

Unfortunately, the interpreter (Arabic speaking) that I got was a female, and she was very uncomfortable talking about it (erectile dysfunction) (Site B_P2_Nurse)

When interpreters were unavailable, respondents sometimes relied on bilingual staff or support person for interpretation as an informal arrangement. Bilingual staff were other health professionals in diverse roles working at the cancer services that also spoke a language other than English and providing language support was not their primary role. Respondents felt that bilingual staff were more impartial than support person/s when interpreting information and had better medical understanding, these were considered advantageous. However, managerial staff raised concerns in the context of patient safety as the language proficiency of bilingual staff in languages other than English was not assessed.

We do use staff occasionally, which I know we're not supposed to but to ‐ just to translate very general things. (SiteA_P12_Nurse)

it can pose risks as well, because there's only the two people involved … we've not tested the language of the health professional or anything. (SiteA_P8_Administrative)

Engagement with family or support person/s was often encouraged for their role as a communicator to raise concerns on behalf of the patient and to relay information to the patient outside of the immediate care appointments/sessions. This role was contingent on support person/s also able to speak and understand English fluently. But concerns were raised regarding completeness and accuracy of information translation when family or support person were used for interpretation.

there is someone who is a close relative or friend of theirs that does speak English and … and they know who to call if the patient is unwell or has any concerns at home. (SiteA_P5_Nurse)

they might express something to their family members later on when they've gone home and then we might get a call from one of the family members. (SiteB_P6_Nurse)

#### 3.1.2 Consumers beliefs of cancer and attitude to engagement

Beliefs and attitudes towards cancer as an illness and related treatment influenced the use of resources to support consumer engagement. Perception of cancer as a shameful disease was identified as a barrier as it impacted the use of interpreters, as often consumers did not want the wider community to know they had cancer. This was particularly identified for smaller ethnic minority communities. Perception of cancer as a terminal illness also impacted the ability of respondents, especially nursing and allied health staff, to engage with patients given the requests made by family or support person/s to not reveal the diagnosis to the patient.

A patient didn't want an interpreter, because they said their community is very small and they don't want people they know to know that they're sick. (SiteA_P6_AlliedHealth)

That may have been because culturally the family didn't want to tell the patient. There was no opportunity to question the passage of information … that had been clearly communicated with the family and the patient, but it hadn't been translated or understood by the patient. (SiteC_P3_Medical)

Consumers' attitudes to engagement were also important as respondents noted that some consumers would want cancer service staff to make decisions for them as staff were considered experts in the field.

Culturally some people may not want to have that same level of decision‐making in their care. They might want someone to say, no, this is the decision… (SiteA_P8_Administrative)

#### 3.1.3 Building trust and knowing more about consumers

Relationships built on trust and knowing more about consumers were highlighted by respondents as enablers for supporting consumer engagement with consumers from ethnic minority backgrounds. Building trust and rapport allowed consumers to feel comfortable to be able to ask questions or raise concerns with more time identified as an essential factor to develop trust.

I think with rapport and trust means that they'll often ask me random questions that aren't related to my role because they trust me and know that I'll find out for them. (SiteD_P5_AlliedHealth)

Staff expressed interest in learning more about the beliefs and perspectives of their patients as individuals to achieve effective engagement. Respondents verbalised the importance of avoiding monoculturalism and viewing patients as individuals to develop personalised strategies.

it's really important not to believe that there's a monocultural approach…. Because I've been caught out many times assuming that I understood what the background influences on a particular person would be. (SiteA_P7_Medical)

Most language diverse patients also have cultural diversity as the added complexity and they tend to have different health views … and that requires a separate discussion on top of the language barrier. (SiteC_P5_Medical)

### 3.2 Tools and technologies support planned communication

Respondents identified two key categories of resources used to bridge the language discordance and to create a common understanding of the care processes and instructions. Resources included media (translated printed/video/audio resources) and technologies (translator applications).

#### 3.2.1 Media (print, video or audio)

Approved translated printed resources were readily available for formal tasks such as the education session (providing general information about cancer treatment) and for high‐risk conditions (e.g., information on management of febrile neutropenia). These resources were often developed by and available through either cancer‐specific health agencies/departments at service/state/federal level. However, there was an overall lack of service‐level resources to support cancer‐specific tasks and care processes. Respondents relied on self‐created cheat sheets or diagrams (such as describing the care task or asking questions) but acknowledged that these were not checked for accuracy. Respondents also pointed towards a general lack of evaluation of resources in the context of patient safety.

We would find that … and print off all the pictures that related so that we would know what they're pointing at and they would see it in their language. (SiteC_P6_Nurse)

Yeah, I can't say we've fully evaluated that (translated information) in that particular (cancer) setting. (SiteA_P11_AlliedHealth)

#### 3.2.2 Technological applications

Respondents, especially nursing and allied health staff, commented on the use and availability of formal (Culturally and Linguistically Diverse [CALD] Assist) and informal applications (Google Translator) to support communication. The key findings regarding the CALD Assist application were that it was not evenly implemented across services, some staff were aware of it and that its focus was on general nursing and allied health tasks (such as assisting with or assessing certain activities of daily living) but did not describe its use in cancer care specific tasks.

we're just slowly pushing that (CALD Assist) through the hospital…, in some places it was working really well, and others, it's taking a bit of time to get it implemented. (SiteA_P8_Administrative)

While the service level policies did not support the use of Google Translator (or similar translator applications), respondents noted frequent use of these applications by consumers and staff.

More staff are using Google Translate. We do have an app on the ward ‐ through the hospital. CALD Assist, it's called. I don't think it's widely used. (SiteD_P12_Nurse)

### 3.3 Organisational policy levers exist but lack implementation in direct care

The ability of service‐level policies and procedures to enable engagement with ethnic minority consumers was directly related to the external system‐level requirements and priorities. Senior managers provided examples of how system‐level requirements shaped local practice, such as the requirement for the use of interpreters for new consultations and during consent processes for clinical trials or initiation of new cancer treatment or change in treatment, but not for a range of other tasks and interactions across the Australian cancer system.

There is a policy on interpreters must be involved in all new patient consults … that are relating to their treatments, consent forms, so when they're consenting to treatment. (SiteB_P1_Administrative)

Policy directives of staff training in cultural competency had resulted in cancer services implementing various cultural training programs, but respondents noted the limitations of such programs to cover a range of cultural practices to enable staff to learn more about each culture. Emerging priority for consumer engagement in healthcare decision‐making was also noted by managers driving efforts at the local level to enhance engagement with consumers generally.

I can see there's a lot more of discussion around patient‐centred care, engaging patients in their care, partnership in care. (SiteA_P8_Administrative)

However, frontline cancer service staff verbalised that the absence or lack of necessary adaptation of current processes for consumer engagement may limit the involvement of consumers from ethnic minority backgrounds in safer care delivery—for example, an administrative staff highlighted that lack of adaption of the current appointment reminder system at their cancer service to meet the needs of low‐English proficient consumers can result in appointment cancellations or inappropriate discharge from cancer care.

COVID‐19 also shaped system‐level practice requirements that in turn influenced opportunities for consumer engagement. Implementation of COVID‐19‐related restrictions meant that interpreters were available through telephone bookings and support person/s was not able to accompany patients during their scheduled appointments and daycare treatment sessions.

Respondents noted that doctors got interpreters more often over the phone than nursing or allied health staff. The wait time for phone interpreters was identified as a consistent issue across the services with respondents noting it being of lower quality than face‐to‐face interpreters. Some allied health and nursing staff verbalised that often phone interpreters were not suitable for their tasks—for example, asking for a particular question that required pointing or describing the physical task. Restrictions for family/support person/s often meant that respondents could not rely on them to confirm the symptoms or relay important patient safety‐related information.

The doctors get the interpreters more so than we do, only because with COVID, it's been really hard with interpreters. (SiteD_P14_Nurse)

A lot of it because of COVID is done not face‐to‐face, so that brings up some barriers … because you don't know what you're saying is being translated effectively. (SiteB_P10_Nurse)

### 3.4 Formal tasks incorporate consumer engagement more readily than informal interactions

Ultimately, the type and degree of formality associated with a clinical or nonclinical task were identified as influencing opportunities for consumer engagement to improve patient safety. Formal tasks enabled opportunities for engagement with consumers from ethnic minority backgrounds because they were more often supported by structures and resources available in the cancer service. Examples of formal tasks include the initial appointment, follow‐up scheduled appointments, initial patient education sessions before chemotherapy or radiotherapy, and obtaining consent for treatment and/or clinical trials.

Formal tasks required healthcare staff to engage with their patients (and sometimes support person/s) in a structured manner to provide the necessary information and ensure the patient's understanding of the instructions provided. Respondents described using interpreters and other resources such as translated printed information sheets to support communication.

Definitely we run an education session … and if they spoke a language other than English as their first language then we would arrange for an interpreter. (SiteA_P5_Nurse)

When we identify someone that's CALD, we actually book interpreters for their first day talks, their first setup on the treatment machine if they speak zero English. (SiteC_P6_Nurse)

Respondents also verbalised using techniques such as speaking slowly, asking follow‐up questions, teach‐back and repeating the information during these formal tasks to engage with patients and ensure their understanding of care. A key factor that supported engagement in these types of tasks was the ability to have planned communication.

I think it's important just to take it a bit slower, make sure that what we're saying to them, they are understanding,… Then we usually have a question time. (SiteA_P2_Nurse)

Would you mind telling me if—repeating back what I've said so I know that you've heard me. (SiteB_P10_Nurse)

While formal tasks in service provision enabled engagement to create safer care, engaging ethnic minority patients during Informal or spontaneous tasks completed in day‐to‐day care was described as more challenging. Informal tasks described included responding to spontaneous requests during patient hospital admission (such as asking about pain or other symptoms), follow‐up treatments sessions in radiotherapy and chemotherapy day care (such as checking for side effects before the start of the treatment session), unscheduled visits and transitions of care (such as booking appointments). In such tasks, cultural and communication support were less readily available to staff and patients, with effective engagement harder to achieve as a result.

After the first session or so, you may not be able to get hold of interpreters and there's a limited supply and resource of them. (SiteB_P3_Nurse)

It's hard to do ward rounds when patients are in‐patients with interpreters. (SiteC_P4_Medical)

Something as simple as the doctor giving them a form to go and have a follow up mammogram. If they don't understand it …. it could impact right down to their morbidity outcome as well. (SiteB_P1_Administrative)

## 4 DISCUSSION

By applying the SEIPS model, this study identified factors that together influence opportunities for engagement with consumers from ethnic minority backgrounds to enhance their safety in the context of cancer services. Service and system‐level features were identified that influenced staff's ability to engage effectively, which predominantly focused on supporting language needs. The degree of formality relative to a healthcare task (e.g., informed consent vs. spontaneous conversation) was associated with the provision of resources to support engagement. Wider service and system‐level policies were key environmental and contextual factors that influenced engagement. Person‐level characteristics such as cultural beliefs, attitudes towards engagement and trust influenced whether someone could engage, desired engagement or required support to engage. Ultimately, where opportunities for consumer engagement were enabled (such as through the use of interpreters or translated resources), an environment that promoted a shared understanding of care processes and goals between clinicians and consumers through strong engagement could be achieved. This study suggests that currently, staff and consumers also face several challenges that inhibit engagement opportunities.

Evidence to date has demonstrated the importance of understanding how safe healthcare is created from a systems perspective. International policy organisations have highlighted the importance of the availability of resources at system and service levels that consistently support consumer engagement for patient safety.^19^, ^37^, ^38^, ^39^ For consumers from ethnic minority backgrounds, inequalities exist in terms of support structures (interpreter availability, time allocated for consultation) that inhibit their ability to access opportunities for engagement that are being steadily created for the general population.^4^, ^15^, ^28^ There is still a long way to go for consumers to gain strong engagement generally, but for CALD communities this challenge is pronounced. The analysis did not clearly identify the physical environment as a factor for consumer engagement despite past earlier research suggesting otherwise.^40^

For consumers from ethnic minority backgrounds, the availability of language support (in the form of interpreters) is imperative for effective consumer‐provider communication.^41^, ^42^ This study identified that often nursing and allied health staff reported communicating with consumers from ethnic minority backgrounds during everyday care interactions (chemotherapy and radiotherapy treatment sessions, ad‐hoc appointments and inpatient cancer care‐related tasks) were not adequately resourced as interpreters were not considered feasible to use or were not available. This finding is important as often during such informal everyday care communications, consumers report safety events they might have experienced, raise concerns or ask questions. While respondents verbalised using bilingual staff occasionally to fill this gap, there is a lack of evidence regarding the use of bilingual staff in the context of patient safety in cancer care. This is also identified as an issue for healthcare settings in general.^4^

Much of the discussion generated through the study focused on language, and cultural factors that influenced engagement were less well understood. The issue of focus on language to assist with consumer–provider communication, while welcomed, does not explicitly address the cultural needs of the ethnic minority population (such as the perception of cancer as a shameful disease impacting interpreter use in some ethnic minority communities). Links between culture, language and patient safety have been explored previously.^4^, ^28^, ^43^ An Australian focus group study with participants from four different ethnic minority population groups identified that cultural factors along with health literacy, English‐language ability and health conditions impact on consumer engagement.^28^ Cultural beliefs about illness, treatment and incongruence of these beliefs between consumers from ethnic minority backgrounds and healthcare providers can also impact consumer engagement for safety.^4^ Overlooking this link between culture, language and safety may also limit the efficacy of system‐level efforts in place to enhance patient safety for ethnic minority populations^4^, ^43^

### 4.1 Implications

Under the right conditions, there are tools available to engage with people from ethnic minority backgrounds to enhance their safety. The current gaps are around systematising these conditions and resources to consistently support safer care in informal and disease‐specific care interactions. This study has two main implications; first, cultural considerations during the development of resources/strategies to support cross‐cultural communication, and second, to support everyday care communication with the use of technology and patient navigators.

Ethnic minority populations are diverse, with considerable intra‐ and interethnic variations.^28^ It has been identified that specific sociocultural beliefs of cancer (such as cancer as a shameful or terminal illness) and related treatment require cultural adaptations for strategies developed to support engagement with consumers from diverse ethnic minority groups.^44^ Particular attention to the cultural meaning of words or phrases used in developing and using language‐specific resources is required.^43^ Cultural considerations during the development of such resources remain important and can be achieved through meaningful involvement of consumers in resource co‐design.^4^, ^43^ One such example is the Making it Meaningful instrument co‐developed with Mandarin‐speaking consumers to enhance their medication safety in cancer care.^45^ This approach could be used as a guide to develop similar resources with consumers from specific ethnic minority groups in specific settings.

Due to the increasing availability of the Internet and technological innovations, translation applications, such as Google Translate, are being used by staff and consumers from ethnic minority backgrounds.^28^, ^46^ While their lack of accuracy in translation has safety implications,^46^, ^47^ their use in the absence of other forms of language support towards enhancing patient safety is not known. A range of health‐specific translator applications (such as MediBabble, xprompt, CALD Assist and Talk to Me) that include preset phrases for different health‐related tasks are now available.^47^ These applications are considered superior to Google Translate in terms of their accuracy.^46^ The translation tools used in general care settings may be insufficient given the importance of the specificity of information that needs to be conveyed in cancer care. In the Australian context, the CALD Assist application has been tested for its usability and acceptability by nursing and allied health staff in the Australian healthcare setting with the studies indicating good acceptability to staff.^41^, ^42^ Future cancer‐specific iteration of the CALD Assist application could be developed but will require caution until further testing is undertaken with consumers from diverse ethnic minority backgrounds.

Studies have established that patient navigation programs are effective in reducing disparity in cancer care outcomes for ethnic minority communities by improving timeliness and access to care.^48^, ^49^, ^50^ Patient navigators (culturally trained healthcare staff, lay people or peers) provide a wide range of support such as scheduling and arranging appointments, providing care co‐ordination, providing communication coaching and assisting with translation to consumers of cancer services^51^ and can assist in enhancing consumer engagement for consumers from ethnic minority backgrounds in their care.^52^ There is potential for targeted implementation and evaluation of programs whereby patient navigators could be trained and used to enable everyday care communications (e.g., daycare treatment procedures or inpatient care interactions).

### 4.2 Limitations

The inclusion of varied healthcare staff from four cancer services across two Australian states provided a comprehensive understanding of a broad range of experiences in a variety of contexts. However, there are certain limitations to the transferability of the study findings. As this study was conducted in cancer services, the application of findings to settings other than cancer may be limited. Further, as this study only explored staff perspectives, there is a need to explore the perspectives of consumers from ethnic minority backgrounds accessing cancer services. This is currently being explored through additional work being conducted. Observation of practice could also be conducted in future research to enhance the triangulation of results. Despite the SEIPS model providing a comprehensive understanding of the range of factors that influence patient safety, some factors that are not linked to patient safety could have been overlooked. Using semistructured interviews may introduce recall bias as participants rely on retelling their experiences but also provide an opportunity to be reflective. The findings should be considered in the context of inter‐ and intraethnic variations as factors identified may vary between and within diverse ethnic minority groups.

## 5 CONCLUSION

Facilitating an environment that promotes a shared understanding of care processes and goals between clinicians and consumers is vital for strong consumer engagement to enhance safety in cancer care for people from ethnic minority backgrounds. This research identified that the infrastructure and resources to support communication with consumers from ethnic minority backgrounds were limited to specific tasks across the cancer care continuum. Strategies implemented by health services to foster effective communication during formal interactions now require expansion to support and create conditions for effective consumer engagement during informal and everyday tasks. The use of innovative language support tools and cultural considerations are required at the service and system level to support consumer engagement in all types of care interactions.

## AUTHOR CONTRIBUTIONS

**Ashfaq Chauhan**: Conceptualization; methodology; formal analysis; validation; project administration; writing—review and editing; writing—original draft; investigation; data curation; resources. **Bronwyn Newman**: Validation; writing— review and editing; data curation; formal analysis; project administration; investigation; supervision. **Elizabeth Manias**: Writing—review and editing; validation. **Kathryn Joseph**: Data curation; validation; formal analysis; writing— review and editing; investigation. **Desiree Leone**: Writing—review and editing; validation. **Ramesh L. Walpola**: Validation; writing—review and editing; supervision. **Holly Seale**: Validation; supervision; writing—review and editing. **Allan Ben Smith**: Validation; writing—review and editing. **Reema Harrison**: Conceptualization; investigation; funding acquisition; writing—review and editing; validation; methodology; supervision; formal analysis; resources; project administration; data curation.

## CONFLICT OF INTEREST STATEMENT

The authors declare no conflict of interest.

## ETHICS STATEMENT

Ethics approval was granted by a National Health and Medical Research Council‐accredited Human Research Ethics Committee (Reference—2020/ETH00965). Written consent was obtained from healthcare staff before data collection.

## Supporting information

Supporting information.

Supporting information.

## Data Availability

Access to the research data from this project may be discussed with the lead author or project lead and is subject to appropriate ethical approvals.

## References

[1] WHO Patient Safety, World Health Organization . *Conceptual Framework for the International Classification for Patient Safety Version 11: Final Technical Report January 2009* (Report No: 606940937X). World Health Organization; 2010.

[2] Hollnagel E , Wears RL , Braithwaite J . From Safety‐I to Safety‐II: A White Paper. The Resilient Health Care Net. University of Southern Denmark, University of Florida, and Macquarie University; 2015.

[3] Runciman W , Hibbert P , Thomson R , Van Der Schaaf T , Sherman H , Lewalle P . Towards an International Classification for Patient Safety: key concepts and terms. Int J Qual Health Care. 2009;21(1):18‐26. 19147597 10.1093/intqhc/mzn057 PMC2638755

[4] Chauhan A , Walton M , Manias E , et al. The safety of health care for ethnic minority patients: a systematic review. Int J Equity Health. 2020;19(1):118. 32641040 10.1186/s12939-020-01223-2 PMC7346414

[5] Lipitz‐Snyderman A , Pfister D , Classen D , et al. Preventable and mitigable adverse events in cancer care: measuring risk and harm across the continuum. Cancer. 2017;123(23):4728‐4736. 28817180 10.1002/cncr.30916 PMC7539686

[6] Weingart SN , Zhang L , Sweeney M , Hassett M . Chemotherapy medication errors. Lancet Oncol. 2018;19(4):e191‐e199. 29611527 10.1016/S1470-2045(18)30094-9

[7] Chauhan A , Chin M , Walpola RL , Seale H , Harrison R , eds. Characterising patient safety events among people from culturally and linguistically diverse backgrounds accessing cancer care in New South Wales: a retrospective medical record review study. NSW Cancer Conference 2023, 2023.

[8] Chauhan A. Consumer engagement for patient safety: Strategies for consumer engagement for culturally and linguistically diverse consumers in cancer services [Internet] . Macquarie University; 2023. Available from: https://figshare.mq.edu.au/articles/thesis/Consumer_engagement_for_patient_safety_Strategies_for_consumer_engagement_for_culturally_and_linguistically_diverse_consumers_in_cancer_services/24023598/1

[9] Carayon P , Bass EJ , Bellandi T , Gurses AP , Hallbeck MS , Mollo V . Sociotechnical systems analysis in health care: a research agenda. IIE Transactions on Healthcare Systems Engineering. 2011;1(3):145‐160. 22611480 10.1080/19488300.2011.619158 PMC3351758

[10] Health Consumers NSW . Who is health consumer? And other definitions. Health Consumers NSW. 2019. Accessed January 24, 2024. https://hcnsw.org.au/training-resources/resources/consumers-toolkit/getting-started/who-is-a-health-consumer-and-other-definitions/

[11] Carayon P , Schoofs Hundt A , Karsh BT , et al. Work system design for patient safety: the SEIPS model. Qual Health Care. 2006;15(suppl 1):i50‐i58. 10.1136/qshc.2005.015842 PMC2464868 17142610

[12] van Rosse F , de Bruijne M , Suurmond J , Essink‐Bot ML , Wagner C . Language barriers and patient safety risks in hospital care. A mixed methods study. Int J Nurs Stud. 2016;54:45‐53. 25840899 10.1016/j.ijnurstu.2015.03.012

[13] Vaismoradi M , Jordan S , Kangasniemi M . Patient participation in patient safety and nursing input ‐ a systematic review. J Clin Nurs. 2015;24(5‐6):627‐639. 25178172 10.1111/jocn.12664

[14] Berglund Kristiansson E , Källman U . Healthcare staff's views on the patients' prerequisites to be co‐creator in preventing healthcare‐associated infections. Scand J Caring Sci. 2020;34(2):314‐321. 31250941 10.1111/scs.12730 PMC7328682

[15] Chauhan A , Walpola RL , Manias E , et al. How do health services engage culturally and linguistically diverse consumers? An analysis of consumer engagement frameworks in Australia. Health Expect. 2021;24(5):1747‐1762. 34264537 10.1111/hex.13315 PMC8483202

[16] Dentzer S . Rx for the ‘blockbuster drug’ of patient engagement. Health Aff. 2013;32(2):202. 10.1377/hlthaff.2013.0037 23381509

[17] Carayon P , Wooldridge A , Hoonakker P , Hundt AS , Kelly MM . SEIPS 3.0: human‐centered design of the patient journey for patient safety. Appl Ergon. 2020;84:103033. 31987516 10.1016/j.apergo.2019.103033 PMC7152782

[18] Holden RJ , Carayon P , Gurses AP , et al. SEIPS 2.0: a human factors framework for studying and improving the work of healthcare professionals and patients. Ergonomics. 2013;56(11):1669‐1686. 24088063 10.1080/00140139.2013.838643 PMC3835697

[19] World Health Organization . Global Patient Safety Action Plan 2021‐2030. WHO; 2021.

[20] Davis RE , Koutantji M , Vincent CA . How willing are patients to question healthcare staff on issues related to the quality and safety of their healthcare? An exploratory study. Qual Saf Health Care. 2008;17(2):90‐96. 18385400 10.1136/qshc.2007.023754

[21] Koutantji M , Davis R , Vincent C , Coulter A . The patient's role in patient safety: engaging patients, their representatives, and health professionals. Clinical Risk. 2005;11(3):99‐104.

[22] Schwappach DLB , Wernli M . Medication errors in chemotherapy: incidence, types and involvement of patients in prevention. A review of the literature. Eur J Cancer Care. 2010;19(3):285‐292. 10.1111/j.1365-2354.2009.01127.x 19708929

[23] Newman B , Joseph K , Chauhan A , et al. Do patient engagement interventions work for all patients? A systematic review and realist synthesis of interventions to enhance patient safety. Health Expect. 2021;24(6):1905‐1923. 34432339 10.1111/hex.13343 PMC8628590

[24] Müller T . Typical medication errors in oncology: analysis and prevention strategies. Onkologie. 2003;26(6):539‐544. 14709927 10.1159/000074148

[25] Weingart SN , Pagovich O , Sands DZ , et al. What can hospitalized patients tell us about adverse events? Learning from patient‐reported incidents. J Gen Intern Med. 2005;20(9):830‐836. 16117751 10.1111/j.1525-1497.2005.0180.x PMC1490203

[26] Weingart SN , Price J , Duncombe D , et al. Patient‐reported safety and quality of care in outpatient oncology. Jt Comm J Qual Patient Saf. 2007;33(2):83‐94. 17370919 10.1016/s1553-7250(07)33010-9

[27] Harrison R , Walton M , Manias E , et al. Codesigning consumer engagement strategies with ethnic minority consumers in Australian cancer services: the CanEngage project protocol. BMJ Open. 2021;11(8):e048389. 10.1136/bmjopen-2020-048389 PMC8330591 34341049

[28] Harrison R , Walton M , Chitkara U , et al. Beyond translation: engaging with culturally and linguistically diverse consumers. Health Expect. 2020;23(1):159‐468. 31625264 10.1111/hex.12984 PMC6978859

[29] Richard JH , Pascale C . SEIPS 101 and seven simple SEIPS tools. BMJ Qual Saf. 2021;30(11):901. 10.1136/bmjqs-2020-012538 PMC8543199 34039748

[30] Tong A , Sainsbury P , Craig J . Consolidated criteria for reporting qualitative research (COREQ): a 32‐item checklist for interviews and focus groups. Int J Qual Health Care. 2007;19(6):349‐357. 17872937 10.1093/intqhc/mzm042

[31] Australian Bureau of Statistics . *Cultural Diversity of Australia: Information on Country of Birth, Year of Arrival, Ancestry, Language and Religion*. ABS; 2022.

[32] Australian Bureau of Statistics . *Data by Region*. ABS; 2023.

[33] Jamshed S . Qualitative research method‐interviewing and observation. J Basic Clin Pharm. 2014;5(4):87‐88. 25316987 10.4103/0976-0105.141942 PMC4194943

[34] The Point of Care Foundation . EBCD: Experienced‐Based Co‐Design Toolkit. Point of Care Foundation; 2018.

[35] Dawda P , Knight A . Experience Based Co‐Design: A Toolkit for Australia. Australian Healthcare & Hospitals Association and Consumer Health Forum of Australia; 2017.

[36] Gale NK , Heath G , Cameron E , Rashid S , Redwood S . Using the framework method for the analysis of qualitative data in multi‐disciplinary health research. BMC Med Res Methodol. 2013;13(1):117. 24047204 10.1186/1471-2288-13-117 PMC3848812

[37] Patient Engagement Action Team . Engaging Patients in Patient Safety—A Canadian Guide. Canadian Patient Safety Institute; 2017.

[38] Australian Commission on Safety and Quality in Health Care . National Safety and Quality Health Service Standard 2: Partnering With Consumers—Embedding Partnerships in Health Care. ACSQHC; 2014.

[39] Alhomoud F , Dhillon S , Aslanpour Z , Smith F . Medicine use and medicine‐related problems experienced by ethnic minority patients in the United Kingdom: a review. Int J Pharm Pract. 2013;21(5):277‐287. 23418849 10.1111/ijpp.12007

[40] Chauhan A , Newman B , Walpola RL , et al. Assessing the environment for engagement in health services: The Audit for Consumer Engagement (ACE) Tool. Wiley; 2022. 10.1111/hex.13610 PMC9700179 36307992

[41] Silvera‐Tawil D , Pocock C , Bradford D , et al. CALD assist‐nursing: improving communication in the absence of interpreters. J Clin Nurs. 2018;27(21‐22):4168‐4178. 29968388 10.1111/jocn.14604

[42] Freyne J , Bradford D , Pocock C , Silvera‐Tawil D , Harrap K , Brinkmann S . Developing digital facilitation of assessments in the absence of an interpreter: participatory design and feasibility evaluation with allied health groups. JMIR Form Res. 2018;2(1):e1. 30684405 10.2196/formative.8032 PMC6334691

[43] Johnstone MJ , Kanitsaki O . Culture, language, and patient safety: making the link. Int J Qual Health Care. 2006;18(5):383‐388. 16956931 10.1093/intqhc/mzl039

[44] Joseph K , Newman B , Manias E , et al. Engaging with ethnic minority consumers to improve safety in cancer services: a national stakeholder analysis. Patient Educ Couns. 2022;105:2778‐2784. 35527113 10.1016/j.pec.2022.04.014

[45] Chauhan A , Newman B , Roberto E , et al. ‘Making it Meaningful’: co‐designing an intervention to improve medication safety for people from culturally and linguistically diverse backgrounds accessing cancer services. Patient Exper J. 2023;10(2):34‐48.

[46] Patil S , Davies P . Use of Google Translate in medical communication: evaluation of accuracy. Brit Med J. 2014;349:g7392. 25512386 10.1136/bmj.g7392 PMC4266233

[47] Panayiotou A , Gardner A , Williams S , et al. Language translation apps in health care settings: expert opinion. JMIR Mhealth Uhealth. 2019;7:e11316. 30964446 10.2196/11316 PMC6477569

[48] Bush ML , Kaufman MR , Shackleford T . Adherence in the cancer care setting: a systematic review of patient navigation to traverse barriers. J Cancer Educ. 2018;33(6):1222‐1229. 28567667 10.1007/s13187-017-1235-2 PMC5711635

[49] Budde H , Williams GA , Winkelmann J , Pfirter L , Maier CB . The role of patient navigators in ambulatory care: overview of systematic reviews. BMC Health Serv Res. 2021;21(1):1166. 34706733 10.1186/s12913-021-07140-6 PMC8555047

[50] Robinson‐White S , Conroy B , Slavish KH , Rosenzweig M . Patient navigation in breast cancer: a systematic review. Cancer Nurs. 2010;33(2):127‐140. 20142736 10.1097/NCC.0b013e3181c40401

[51] Chan RJ , Milch VE , Crawford‐Williams F , et al. Patient navigation across the cancer care continuum: an overview of systematic reviews and emerging literature. CA Cancer J Clin. 2023;73:565‐589. 37358040 10.3322/caac.21788

[52] Brady B , Sidhu B , Jennings M , et al. The feasibility of implementing a cultural mentoring program alongside pain management and physical rehabilitation for chronic musculoskeletal conditions: results of a controlled before‐and‐after pilot study. BMC Musculoskelet Disord 2023;24(1):47. 36658511 10.1186/s12891-022-06122-x PMC9850562

