# Correction to “Creating Safer Cancer Care With Ethnic Minority Patients: A Qualitative Analysis of the Experiences of Cancer Service Staff”

**DOI:** 10.1111/hex.70402

**Published:** 2025-08-21

Chauhan A, Newman B, Manias E, Joseph K, Leone D, Walpola RL, Seale H, Smith AB, Harrison R. Creating safer cancer care with ethnic minority patients: A qualitative analysis of the experiences of cancer service staff. Health Expect. 2024 Feb;27(1):e13979. doi: 10.1111/hex.13979.

The name of the eighth author is misspelled. It should be spelled as ‘Allan with a’ instead of ‘Allen with e’. Can we please request to change this to ‘Allan’.

We apologize for this error.

