# Supplementary material for: Creating safer cancer care with ethnic minority patients: A qualitative analysis of the experiences of cancer service staff
Source: Health Expect. 2024 Jan 30;27(1):e13979. doi: 10.1111/hex.13979 (PMC10825879; doi:10.1111/hex.13979)
Supplement: Supplementary file 2 — Supporting information. [file HEX-27-e13979-s001.docx]

**Supplementary File B: Framework Matrix**

| **SEIPS Category (definition)** | **Subthemes coded** | **Theme identified** |
| --- | --- | --- |
| **Person:** either patient or health staff, interaction/relationship between staff and consumers; or demographics | People (interpreters, bilingual co-workers and care support person) supporting communication.  Building relationships and trust  Cultural understanding of illness and treatment  Decision making and choice.  Staff skills and awareness. | *Consumer- service provider dyad* |
| **Tools and Technologies:** strategies used to enhance communication and engagement. | Translated information.  Technological applications and communication resources. | *Resources (tools and technologies) to support consumer engagement for safety.* |
| **Tasks:**  processes of care and how engagement occurred in those specific activities. | Providing education and taking consent  Follow up treatment and other appointments. | *Formal tasks incorporate consumer engagement more readily than informal interactions* |
| **Organisational conditions:** organisational level resources or conditions that impact engagement. | Interpreter availability  Staff training  Cultural Awareness  Governance  Systems | *Impact of organisational and broader context on consumer engagement for patient safety* |
| **External environment:** | COVID 19  System level policies |  |
| **Internal physical environment** |  | *Not identified* |
